# Identification of a Novel Modulator of Thyroid Hormone Receptor-Mediated Action

**DOI:** 10.1371/journal.pone.0001183

**Published:** 2007-11-21

**Authors:** Bernhard G. Baumgartner, Meritxell Orpinell, Jordi Duran, Vicent Ribas, Hans E. Burghardt, Daniel Bach, Ana Victoria Villar, José C. Paz, Meritxell González, Marta Camps, Josep Oriola, Francisca Rivera, Manuel Palacín, Antonio Zorzano

**Affiliations:** 1 Institute for Research in Biomedicine (IRB Barcelona) and Departament de Bioquímica i Biologia Molecular, Facultat de Biologia, Universitat de Barcelona, Barcelona, Spain; 2 Servei Hormonal, Hospital Clinic i Provincial, Barcelona, Spain; Canadian Agency for Drugs and Technologies in Health, Canada

## Abstract

**Background:**

Diabetes is characterized by reduced thyroid function and altered myogenesis after muscle injury. Here we identify a novel component of thyroid hormone action that is repressed in diabetic rat muscle.

**Methodology/Principal Findings:**

We have identified a gene, named *DOR*, abundantly expressed in insulin-sensitive tissues such as skeletal muscle and heart, whose expression is highly repressed in muscle from obese diabetic rats. DOR expression is up-regulated during muscle differentiation and its loss-of-function has a negative impact on gene expression programmes linked to myogenesis or driven by thyroid hormones. In agreement with this, DOR enhances the transcriptional activity of the thyroid hormone receptor TR_α1_. This function is driven by the N-terminal part of the protein. Moreover, DOR physically interacts with TR_ α1_ and to T_3_-responsive promoters, as shown by ChIP assays. T_3_ stimulation also promotes the mobilization of DOR from its localization in nuclear PML bodies, thereby indicating that its nuclear localization and cellular function may be related.

**Conclusions/Significance:**

Our data indicate that DOR modulates thyroid hormone function and controls myogenesis. *DOR* expression is down-regulated in skeletal muscle in diabetes. This finding may be of relevance for the alterations in muscle function associated with this disease.

## Introduction

Thyroid hormones play a central role in metabolic homeostasis, development, cell differentiation and growth [1]–[3]. Disorders in thyroid function are among the most common endocrine diseases and affect 5–10% of individuals during their lifetime [4]. Thyroid hormones stimulate basal metabolic rate and adaptive thermogenesis through effects on major metabolic tissues such as skeletal muscle, liver and adipose tissue. The major effects of thyroid hormones are mediated by modulation of gene transcription. Most thyroid response elements function in such a way that thyroid hormone receptors (TRs) repress gene transcription in the absence of ligand and are activated after binding to thyroid hormones. In the presence of T_3_, TR undergoes a conformational change which results in the replacement of a co-repressor by a co-activator complex, which in turn triggers the transcriptional activation of TR-regulated genes.

Thyroid hormone response elements have been identified in muscle-specific genes such as *myogenin*, *α-actin*, or *GLUT4* [5]–[7]. Several TR-regulated genes determine distinct aspects of muscle biology. Thus, thyroid hormones regulate muscle development and function by inducing myoblast cell cycle exit [8]. In addition, thyroid hormones exert substantial effects on myotube formation and muscle fiber composition by regulating the expression of several masters of differentiation such as MyoD or myogenin [9]–[12] or by inducing muscle-specific genes such as the *myosin heavy chain* [13], [14]. Thyroid hormones also affect the outcome of repair in adult muscle. Thus, conditions of increased circulating T_3_ levels are characterized by a shortening of the time in which myoblasts are in a proliferative state and by speeding up their transition to fusion; this pattern of changes reduces the number of myotubes that are produced during injury repair [15]. In contrast, hypothyroidism slows myoblast proliferation and reduces the number of new myotubes formed during repair [16].

Here we identified a novel protein, DOR, which is abundantly expressed in insulin-sensitive tissues and it is markedly repressed in diabetes. We also report that DOR regulates thyroid hormone action. Taken together, our data suggest that DOR determines muscle development, function and metabolic response to hormonal cues through modulation of the expression of TR-regulated genes.

## Results

### Identification of *DOR*, a gene that is abundantly expressed in skeletal muscle and heart and is down-regulated in obese diabetic rats

To identify potential risk factors for the alterations associated with type 2 diabetes, we screened genes differentially expressed in Zucker diabetic fatty (ZDF) rats and non-diabetic lean rats (control) by PCR-select cDNA subtraction. After obtaining the subtracted cDNA library, we isolated several clones using differential screening by PCR-selection. One of these clones was chosen and used as a probe, which further allowed the detection of a 4.5 kb mRNA species in various tissues. A human heart cDNA library was then screened and the full-length cDNA of human *DOR* was isolated. This cDNA coincided with the predicted open reading frame C20orf110 (NM021202). Given the criteria that led to its identification, we named the gene *DOR* for Diabetes- and Obesity Regulated. Human *DOR* maps to chromosome 20q11.22, close to loci linked to human obesity [17]–[19] and type 2 diabetes [20]–[22]. A comparison between the genomic and the cDNA product reveals an intronic-exonic distribution of four introns and five exons. The protein coding region starts at exon 3 and generates an ORF of 672 nucleotides. Murine and rat *DOR* cDNAs were also amplified and sequenced.

**Figure 1:**
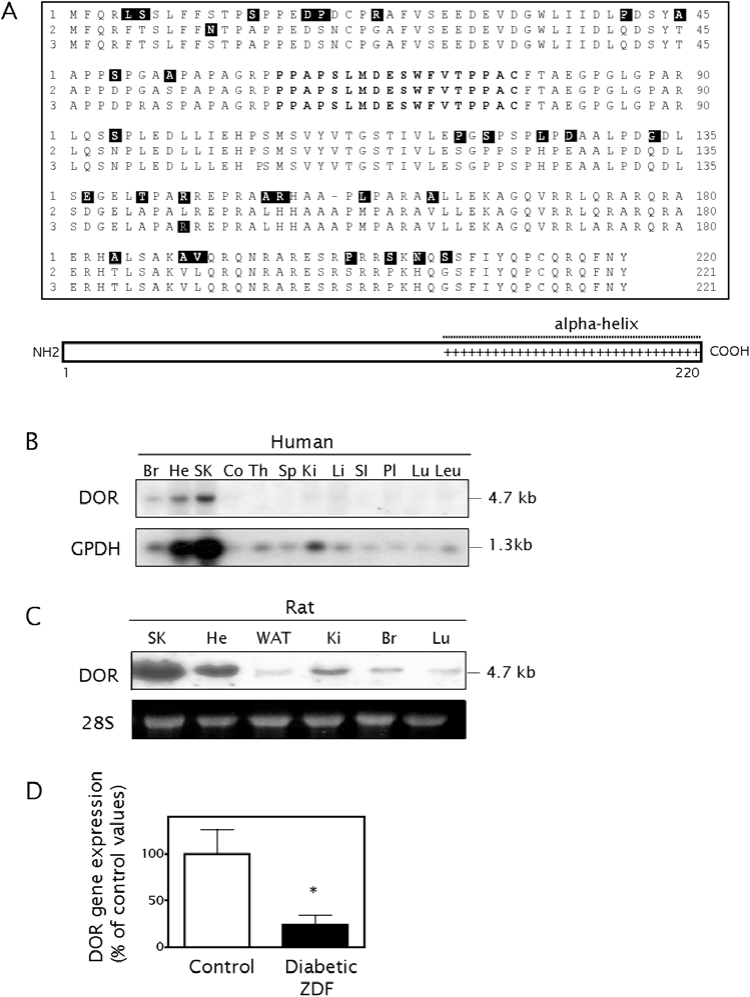
DOR sequences and tissue distribution of *DOR* expression and down-regulation in skeletal muscle from ZDF rats. Panel A. Amino acid sequence of human, mouse and rat DOR proteins (sequences 1, 2 and 3, respectively). Multi-alignment done using the CLUSTALW Sequence Alignment programme [52]. Amino acids differing from the consensus are inverse. The amino acid residues used to generate the polyclonal antibodies are shown in bold. The C-terminal basic motif, indicated by a line of “+”, is predicted to form an alpha-helix structure whereas the N-terminal half is unstructured (GLOBPLOT 2). Panel B. PolyA^+^-RNA membrane containing human adult tissues was probed with ^32^P-labelled rat *DOR* cDNA and washed in stringent conditions. The probe hybridises to a transcript of approximately 4.5 kb. Hybridisation with human glycerol-3-phosphate dehydrogenase (GPDH) cDNA was used as a control probe. Br, brain; He, heart; SK, skeletal muscle; Co, colon; Th, thymus; Sp, spleen; Ki, kidney; Li, liver; Sl, small intestine; Pl, placenta; Lu, lung; Leu, leukocytes. Panels C. Total RNA was purified from several rat tissues and subjected to Northern blot analysis. Ethidium bromide staining of the ribosomal 28S subunit was used as a control of the relative amounts of RNA loaded in each lane and to check the integrity of RNA in each sample. SK, skeletal muscle; He, heart; WAT, white adipose tissue; Ki, kidney; Br, brain; Lu, lung. Panel D. Total RNA was purified from skeletal muscle from non-diabetic and ZDF rats, and RNA was subjected to Northern blot analysis. The mean±SD of 6 separate observations is shown. * difference compared to the control group, at P<0.01 (Student's *t* test).

Human, rat and mouse DOR polypeptides are well conserved (84% identity between human and mouse, 83% between human and rat, 85% between rat and mouse), and encode a protein of 220 (human) or 221 (mouse, rat) residues (Figure 1A). The only homologous protein described to date is a human p53-dependent apoptosis regulator named p53DINP1/TEAP/SIP, with 36% identity with human DOR [23]. DOR contains a strong positive charge in its C-terminal region, which is predicted to form an alpha-helix structure (Figure 1A) whereas the rest of the protein is predicted to be unstructured (GLOBPLOT 2) [24].

The distribution of *DOR* mRNA in human and rat tissues was examined by Northern blot. In the two models, transcripts were predominant skeletal muscle and heart, while lower expression was detected in other tissues such as white fat, brain, kidney and liver (Figure 1B, 1C and data not shown). These data suggest a potential role of DOR in tissues with high metabolic requirements or which respond to insulin. We also examined *DOR* expression in skeletal muscle from ZDF rats and found a reduction of 77% in its expression (Figure 1D), thereby corroborating the original subtraction hybridization assay.

Using differential screening, we thus identified a novel protein which is strongly repressed in obese diabetic rats, and highly expressed in tissues involved in metabolic homeostasis. Next, we analyzed DOR cellular function in order to determine the contribution of alterations in *DOR* expression to the pathophysiology of diabetes.

### DOR is a nuclear protein that enhances the activity of TRs

**Figure 2:**
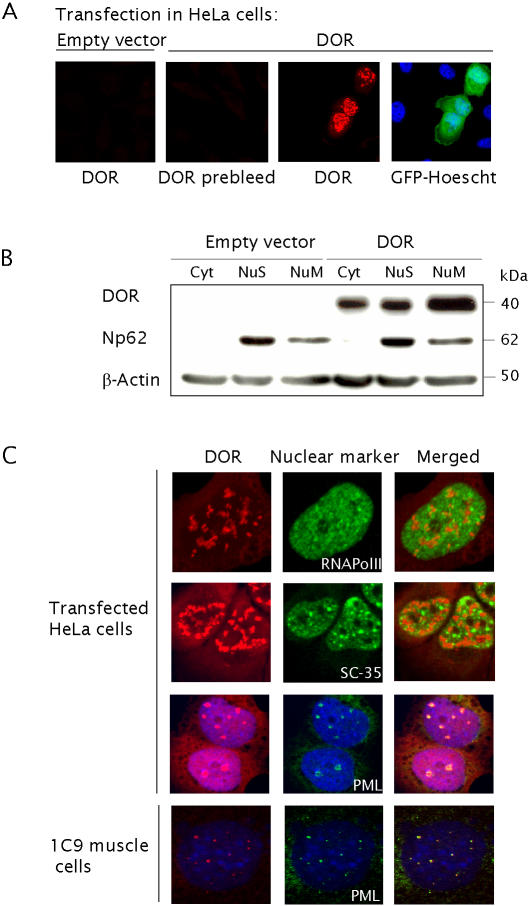
DOR protein is localized in nuclear bodies. Panel A. HeLa cells were transfected with a DOR expression vector or with the empty vector and with GFP. After 48 h, cells were fixed and stained to view DOR, GFP or with the DOR pre-immune serum (negative control). Cells were also counterstained with Hoescht. The arrow indicates a GFP-positive cell, also DOR-positive. DOR shown in red; GFP in green; nuclei counterstained in blue. Panel B. After DOR transfection in HeLa cells (48 h), cytosolic (Cyt), nuclear soluble (NuS) or nuclear nonsoluble (NuM) fractions were obtained by an osmotic-shock method. Fractions were subjected to Western Blot assays with anti-DOR, anti-Np62 or anti- β-actin antibodies. Panel C. DOR-transfected HeLa cells or wild-type mouse 1C9 myoblasts were fixed and stained to view DOR and markers of subnuclear domains, such as the splicing speckles (SC-35), PML bodies (PML) or transcriptionally active sites (RNA Pol. II). DOR is shown in red in the images on the left, and SC-35, PML or RNA Polymerase II are shown in green in the middle. Merging is shown on the right.

Several lines of evidence support the notion that DOR has a nuclear function, namely: a) DOR is predicted to be a nuclear protein (WoLF PSORT Prediction programme) [25], and b) DOR uniquely shows homology to a nuclear protein. To test the first hypothesis, HeLa cells were transfected with a plasmid encoding DOR ORF and the protein was detected by Western Blot or immunofluorescence. DOR migrated as a 40 kDa protein in SDS-PAGE (Figure 2B). By subcellular fractionation assays, DOR was detected in nuclear extracts (Figure 2B). Immunofluorescence data confirmed this observation since DOR was localized mainly in nuclei (Figure 2A). Within the nucleus, DOR colocalized with PML bodies (Figure 2C). This colocalization was not due to the over-expression of DOR in this cell line since the endogenously expressed protein was also detected in these PML nuclear bodies in murine 1C9 muscle cells (Figure 2C) derived from the immortomouse [26].

**Figure 3:**
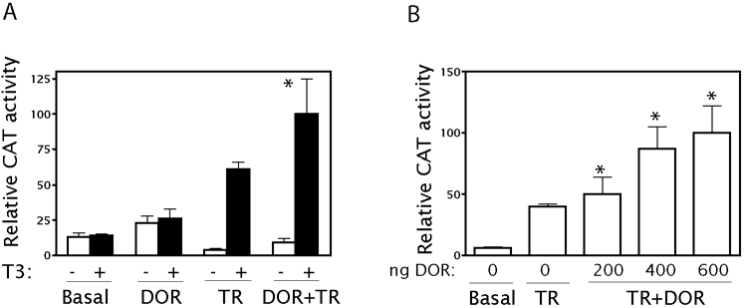
DOR transactivates nuclear hormone receptors. Panel A. HeLa cells were transfected with expression plasmids encoding TR_α1_ (TR), DOR, the empty vector pcDNA3 as a control vector, and the reporter vectors containing TR_α1_ response elements linked to CAT. Cells were treated for 18 h in the presence or absence of ligands (100 nM T_3_) and assayed for reporter expression. Results are mean±SD of 6 independent experiments. * significant difference compared to the nuclear hormone receptor group, at P<0.05 (post hoc *t* test). Panel B. Reporter assays were done as in previous panels but the amounts of DOR (ranging from 200 to 600 ng) used for transfection differed and these assay were done in the presence of ligands. Results are mean±SD of 6 independent experiments. * significant difference compared to the nuclear hormone receptor group, at P<0.05 (post hoc *t* test).

DOR was localized within the nucleus, thereby corroborating the theoretical prediction. On the basis of this observation, and given that DOR is homologous to a nuclear protein involved in transcriptional regulation, we proposed that it also regulates transcription. Furthermore, the high DOR expression in tissues characterized by high metabolic requirements led us to speculate about a regulatory role of this protein in thyroid hormone action. To this end, HeLa cells were transfected with DNA-encoding TR_α1_ and CAT or luciferase reporters gene fused to TR elements, in the presence or absence of DOR. TR_α1 _transactivated the reporter gene, whereas DOR alone showed a small stimulatory effect on reporter activity (Figure 3A). The cotransfection of DOR and TR_α1_ enhanced the transcriptional activity of the reporter gene in a dose-dependent manner (Figures 3A, 3B). This effect was specific of DOR expression and transfection with a plasmid encoding the xCT amino acid transporter did not cause any effect (data not shown). The effects of DOR were also detected when using *luciferase* as a reporter gene (data not shown). DOR did not cause any effect on the reporter activity induced by transcription factors p53 or c-Myc (data not shown). In addition, DOR did not stimulate the activity of the chimeric protein GAL4-VP16, generated by fusion of the GAL4 DNA-binding domain and the VP16 activation domain (data not shown). In all, these observations indicate that DOR specifically potentiates the activity of TRs. The effect of DOR is not a consequence of a generalized stimulation of transcription since basal reporter activity, activity driven by c-Myc or p53, and activity of GAL4-VP16 remained unaltered.

**Figure 4:**
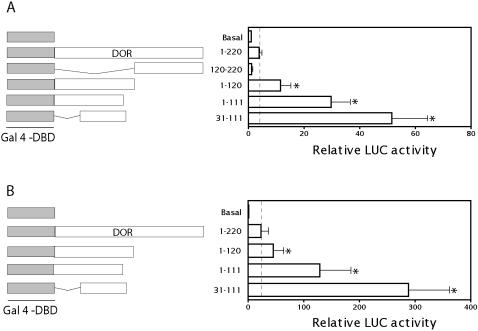
DOR shows transcriptional activity when tethered to a target gene promoter. DOR or fragments corresponding to the amino acids indicated were fused to the DNA-binding domain of Gal4 (Gal4 DBD) and transfected in HeLa cells (panel A) or in HEK293T cells (panel B). Transcription was assayed with a reporter plasmid containing five copies of the UAS linked to luciferase. Results are mean±SD of 6 independent experiments. * difference compared to the Gal4 DBD-DOR group, at P<0.05 (post hoc *t* test).

To analyze whether DOR acts as an activator when tethered to DNA, full-length DOR or distinct cDNA fragments were fused to a GAL4 DNA-binding domain (Gal4-DBD) and transcriptional activity was assayed by cotransfection with a Gal4 reporter plasmid in HeLa cells. Gal4-DBD fused to full-length DOR caused a moderate increase (3-fold) in reporter activity (Figure 4A) and deletion of the C-terminal half of the protein (fragment 1–120) markedly increased this activity (8.5-fold) (Figure 4A). The fragment encompassing amino acid residues 31–111 showed the maximal stimulatory activity (47-fold) (Figure 4A). In contrast, the C-terminal half of DOR showed no transcriptional activity. Similar data were obtained in HEK293T cells (Figure 4B). These data suggest that the N-terminal half of DOR shows transcriptional activity, and this activity is increased when the C-terminal half of the protein is deleted.

### DOR loss-of-function reduces the action of thyroid hormones in muscle cells

**Figure 5:**
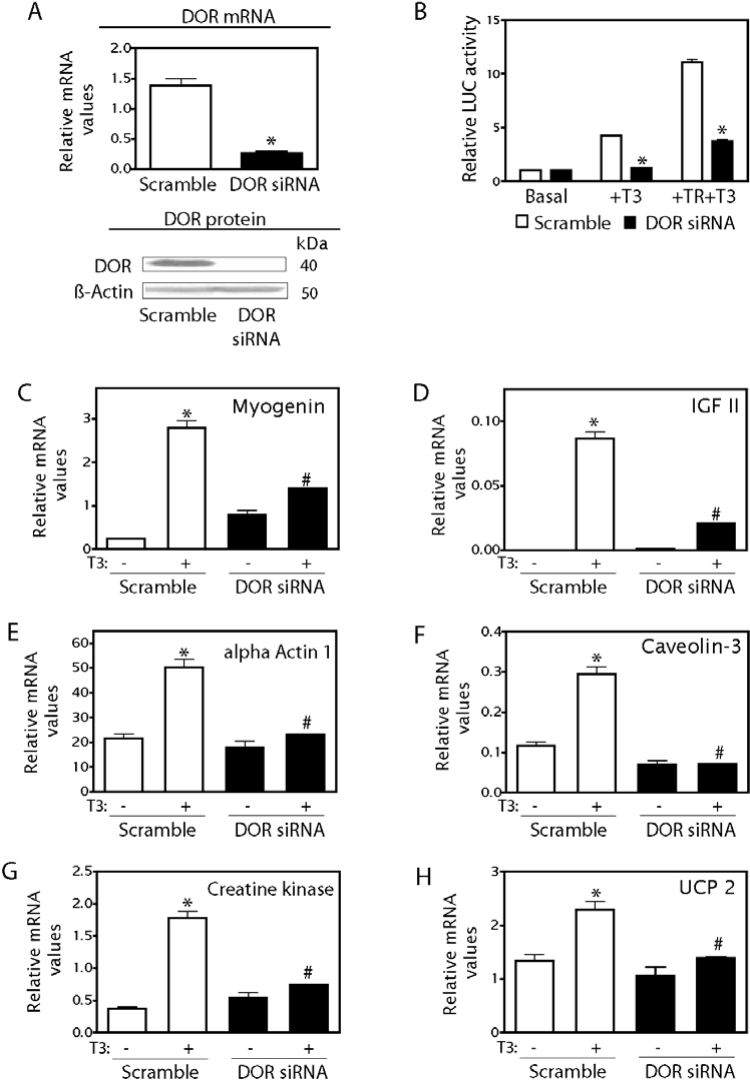
DOR loss-of-function in muscle cells. Panel A. C2C12 myoblasts previously infected with lentiviruses encoding scrambled RNA (open bar) or DOR siRNA (black bar) were cultured. Cell extracts and total RNA were obtained and DOR protein and mRNA levels were assayed by Western blot and real-time PCR. Relative amounts of proteins in each sample were checked by expression of the nonmuscle-specific protein β-actin. * difference compared to the scrambled group, at P<0.05 (Student's *t* test). Panel B. Scramble (open bars) or DOR siRNA C2C12 muscle cells (black bars) were transfected with a reporter vector driven by a TRE, and with or without a expression vector for TR_α1_. Cells were then incubated in the presence or absence of thyroid hormone for 16 h. Results are mean±SD of triplicates and are representative of three independent experiments. * difference compared to the scrambled group, at P<0.05 (post hoc *t* test). Panels C–H. Scrambled (open bars) or DOR siRNA C2C12 muscle cells (black bars) were incubated in 5% horse serum-containing medium either in the absence or in the presence of 100 nM T_3_. Total RNA obtained at 48 h of T_3 t_ treatment were assayed by real-time PCR to measure the expression of several genes. Results are mean±SD of a representative experiment. * difference compared to the control group, at P<0.05 (post hoc *t* test).

To determine whether DOR is required for thyroid hormone action, we generated lentiviral vectors encoding for siRNA to knock-down (KD) DOR expression in mouse cells. The siRNA lentiviral infection in C2C12 muscle cells markedly reduced DOR expression (80% reduction) compared to levels found in cells infected with scrambled RNA (control group) (Figure 5A). Once the KD system had been validated, control and KD cells were transiently transfected with a reporter gene driven by a TRE, in the presence or absence of TR_α1 _or T_3_. In control muscle cells, while thyroid hormone caused a 5-fold stimulation of reporter activity through the activation of endogenous TR_ α1_, the addition of exogenous TR increased the stimulation of transcriptional activity up to10-fold (Figure 5B). DOR loss-of-function markedly reduced the effect of T_3_, TR_α1 _and T_3_ (Figure 5B).

On the basis of these data, we next tested whether the reduced DOR expression altered the effect of thyroid hormones on endogenous target genes. In control C2C12 muscle cells, stimulation with T_3_ (100 nM for 48h) markedly enhanced the expression of genes such as *myogenin*, *IGF-II*, *actin α1*, *caveolin-3*, *creatine kinase* and *UCP2* (Figure 5 C–H). Stimulation of *α-actin* and *myogenin* expression in response to thyroid hormones has been previously reported [10], [11]. Under these same conditions, DOR-KD cells markedly reduced the stimulatory effect of thyroid hormones on the expression of the same subset of genes (Figure 5 C–H).

On the basis of these data, and the previous observation that DOR enhances the transcriptional activation of TR_α1_ (Figure 3), we propose that DOR regulates TR-mediated cellular responses.

### Functional role of DOR in myogenic differentiation

**Figure 6:**
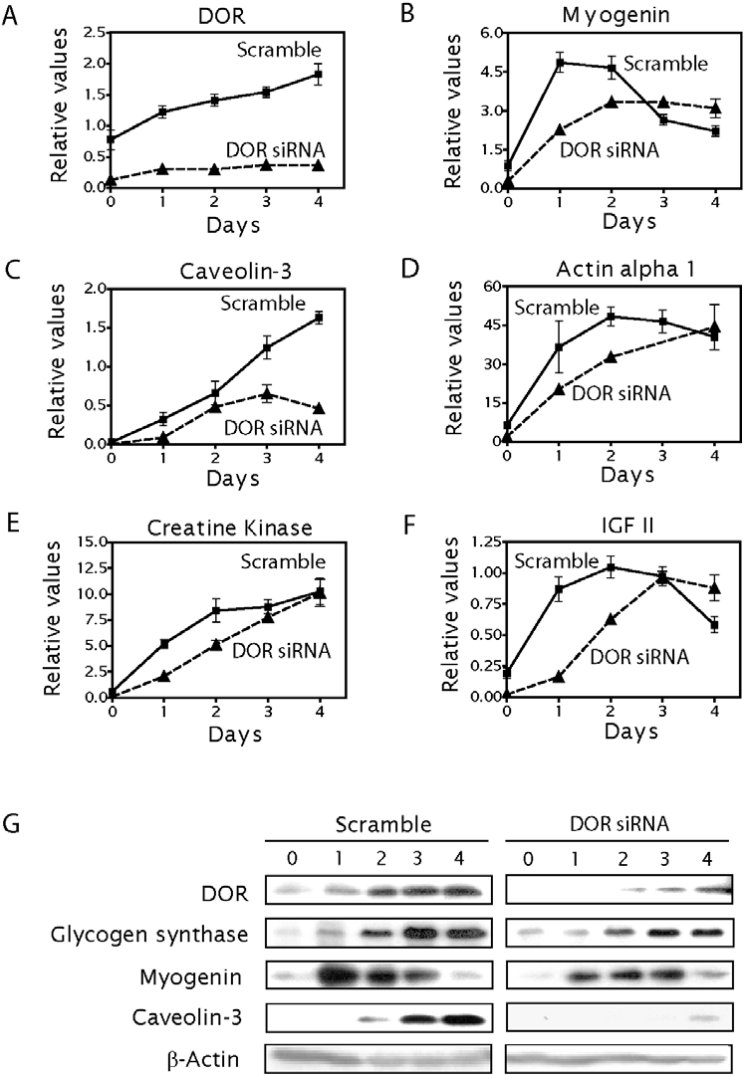
DOR loss-of-function alters myogenesis. Panels A–F. Confluent C2C12 myoblasts previously infected with lentiviruses encoding scrambled RNA (squares) or DOR siRNA (triangles) were allowed to differentiate in 5% horse serum-containing medium for 4 days. Total RNA was purified and the expression of *DOR*, *myogenin*, *caveolin-3*, *actin α1*, *creatine kinase*, *IGF-II* and *HPRT* was assayed by real-time PCR. Values were expressed as relative to *HPRT*. Results are mean±SD of four independent experiments. Scrambled and DOR siRNA groups were significantly different as analyzed by two-way ANOVA, at P<0.05. Panel G. DOR and muscle-specific protein expression (myogenin, caveolin 3, and glycogen synthase) were analyzed by Western blot of total cell lysates (20 µg) from each condition. Relative amounts of proteins in each sample were checked by expression of the nonmuscle-specific protein β-actin.

Given that DOR expression is markedly repressed in muscle from ZDF rats and that diabetes is linked to skeletal muscle atrophy [27]–[30], we next studied whether DOR participates in myogenesis. To this end, the expression of several genes and proteins in scrambled or DOR siRNA C2C12 cells was examined during myogenic differentiation (from myoblasts to myotubes). Muscle differentiation in C2C12 cells caused a 3-fold stimulation of *DOR* expression (Figure 6A), which was blocked in DOR KD cells (Figure 6A). During C2C12 myoblast differentiation, several muscle-specific genes such as *myogenin*, *creatine kinase*, *caveolin 3*, *actin α1* and *IGF-II* were markedly induced in control cells (from 10- to 20-fold) (Figure 6 B–F). Under these conditions, DOR-KD cells showed altered induction in the expression of these genes (Figure 6 B–F). However, the expression pattern of each gene differed. Myogenin, a transcription factor which plays a unique function in the transition from a determined myoblast to a fully differentiated myotube [31], [32], was rapidly induced in early stages of differentiation. While control cells normally induced myogenin mRNA levels (5-fold stimulation at day 1 of differentiation), DOR-KD cells showed a delay (Figure 6B). However, at day 3 of differentiation, no differences were detected between control and KD cells (Figure 6B). For *actin α1*, *creatine kinase* and *IGF-II*, the inhibition of expression was greater at the onset of differentiation (days 1 and 2) (Figure 6 D–F). The expression of caveolin-3 was greatly repressed during differentiation in DOR-KD cells (Figure 6C). Finally, the expression of muscle-specific genes at the protein level was also analyzed. Our findings further confirmed that DOR siRNA reduced the abundance of myogenin, glycogen synthase or caveolin-3 compared to control cells (Figure 6 G).

In all, our results indicate that DOR plays a regulatory role in the myogenic programme, and more specifically, during early stages of muscle differentiation.

### DOR physically binds TR_α1_

**Figure 7:**
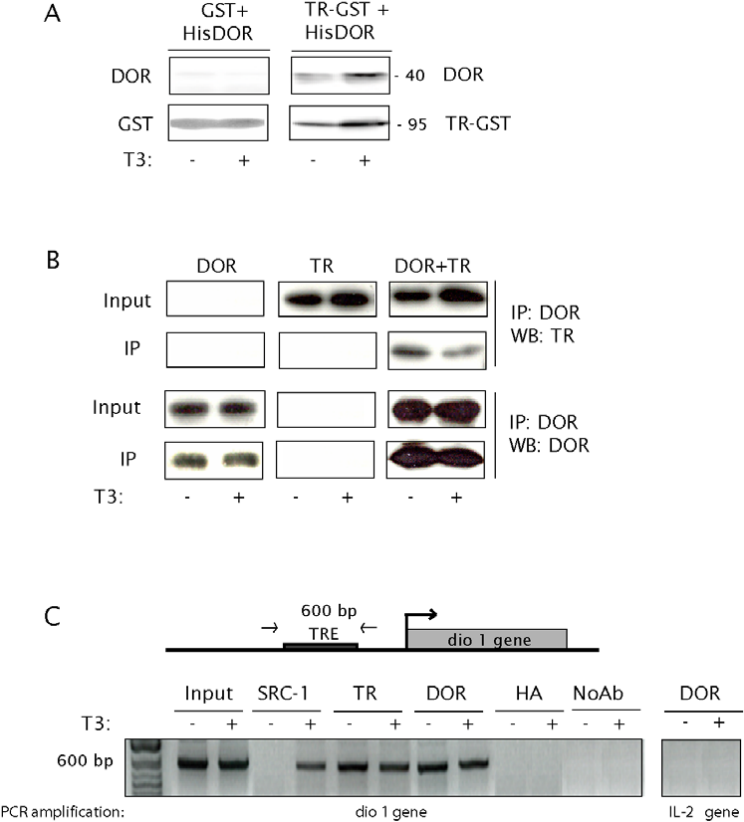
DOR binds *in vitro* and *in vivo* to thyroid hormone receptors. Panel A. GST protein or TR_α1_ fused to GST (TR-GST) were immobilized on glutathione sepharose beads and incubated with the DOR protein containing an N-terminal histidine tag (HisDOR), with or without the ligand (1 µM T_3_). Bound proteins were eluted and resolved by SDS-PAGE and further Western blot using an antibody against the histidine tag (to visualize HisDOR) or against GST (to visualize GST or TR-GST). Panel B. HeLa cells over-expressing His-tagged DOR (left), TRα-1 (middle), or both (HisDOR+TR_α1_) (right) were exposed to T_3_ or were left untreated. After 1 h of treatment, cells were collected and DOR was immunoprecipitated from the nuclear fractions. The input control (10% input) and the immunoprecipitates (IP) were assayed by Western blot with specific antibodies. Panel C. ChIP analysis over a T_3_ responsive promoter. DOR and TR_α1_-transfected HeLa cells were treated with T_3_ for 1 h or left untreated. Cross-linked chromatin prepared from cells was immunoprecipitated with the antibodies indicated. As negative controls, the samples were subjected to ChIP in the absence of antibody or in the presence of an irrelevant antibody (anti-hemaglutinin, HA). Aliquots of chromatin taken before immunoprecipitation (input) and the immunoprecipitates were subjected to PCR analysis with primers directed to the dio1 promoter. DOR immunoprecipitates were used to amplify *IL-2* (an additional negative control group).

On the basis of the observation that DOR functionally modulates thyroid hormone action, we also examined whether DOR and TRs physically interact. To this end, chimeric fusion proteins TR_α1_-GST, RXR-GST, and DOR-His were produced. TR_ α1_-GST bound DOR protein and the physical interaction in pull-down assays was independent of the presence of T_3_ in the medium (Figure 7A). Under these conditions, neither GST nor RXR-GST bound DOR protein (Figure 7A and data not shown). To verify that the DOR-TR_ α1 _interaction was also established *in vivo*, HeLa cells were transfected with DOR, TR_ α1_ or both, in the presence or absence of T_3_, and extracts were immunoprecipitated with an anti-DOR antibody. The bound proteins were eluted and analyzed by Western blot with polyclonal antibodies against TR_ α1_ or DOR. We detected specific co-immunoprecipitation of TR_ α1_ and DOR proteins both in the presence and absence of T_3_ (Figure 7B).

Next, we determined whether this binding also occurred *in vivo* in the context of a T_3_-responsive promoter of a gene transcribed in HeLa cells. Thus, we selected the human *dio1* gene promoter, since its mRNA is expressed in this cell line [33]. DOR-TR_ α1_-transfected HeLa cells, treated or not with T_3_ for 1 h, were subjected to ChIP assays by using DOR, TR_ α1_ or SRC-1 antibodies. The resulting precipitated genomic DNA was then analyzed by PCR using primers flanking the boundaries of the TREs located in the promoter region of *dio1* [34]. Under these conditions, SRC-1 was recruited in the complex only after T_3_ treatment (Figure 7C), while TR_ α1_ was bound both in the presence and absence of T_3_ (Figure 7C). The same pattern was detected with antibodies against DOR (Figure 7C), thus confirming the results obtained by Co-IP. ChIP assays in the absence of antibodies did not amplify any unspecific band (Figure 7C). DOR immunoprecipitates did not amplify a fragment of *interleukin-2*, used as a negative control (Figure 7C). Similarly, immunoprecipitates with an irrelevant antibody (anti-hemaglutinin, HA) did not amplify *dio1* (Figure 7C)

In all, we observed either by CoIP or ChIP methods that DOR physically binds TR in a ligand-independent manner, while the functional activation is ligand-dependent. On the basis of these data, we hypothesize that the presence of other proteins of the TR complex ultimately determine DOR function.

### Thyroid hormones rapidly modulate the intranuclear localization of DOR

**Figure 8:**
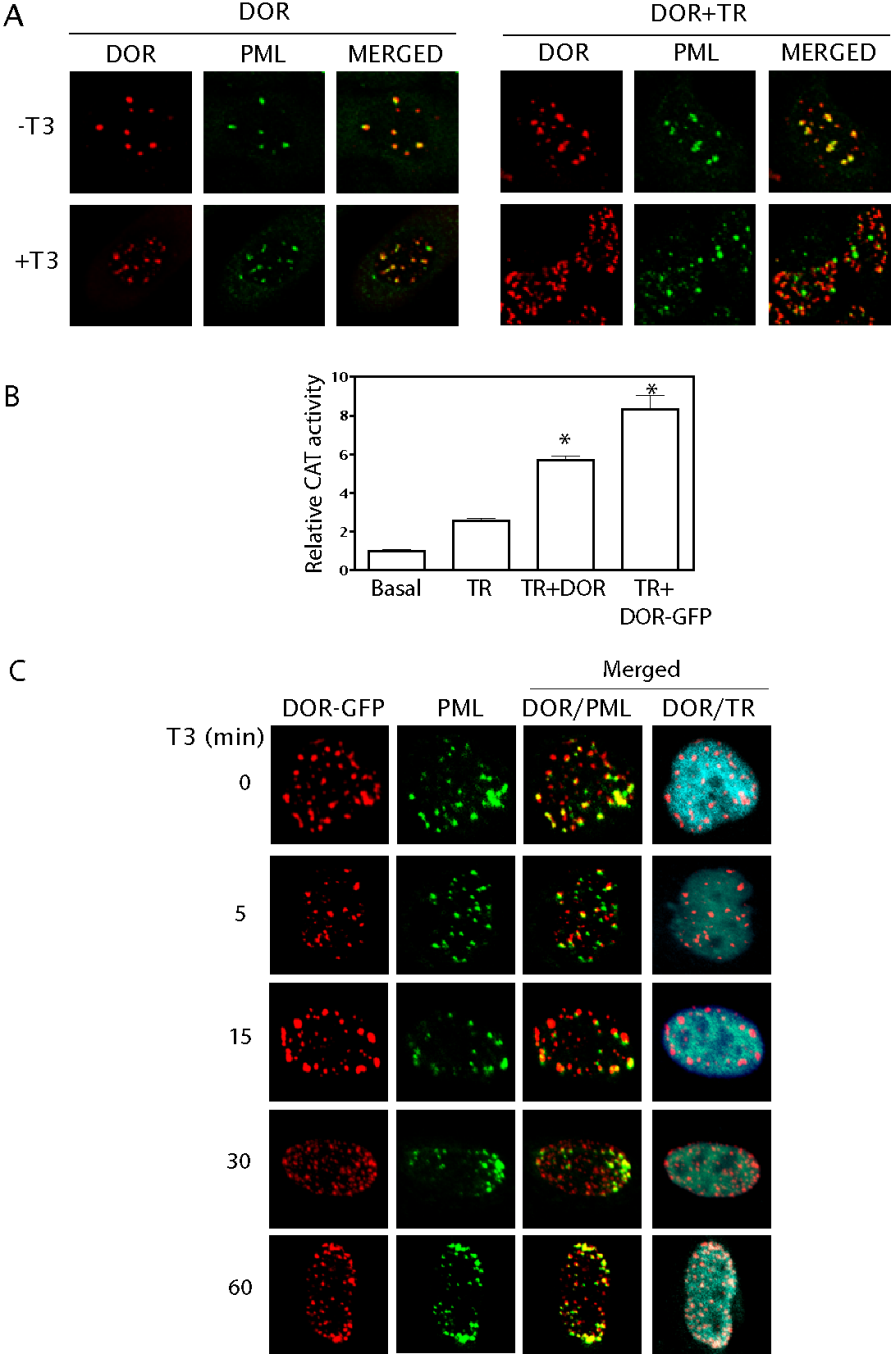
DOR rapidly delocalizes from PML nuclear bodies in response to thyroid hormones. Panel A. HeLa cells were transiently cotransfected with DOR and TR_α1_ expression vectors. The intranuclear positioning of DOR relative to PML nuclear bodies was determined before and after T_3_ addition. Antibodies and immunofluorescence legend: Anti-DOR, stained red (column 1); anti-PML, stained green (column 2); merged images (column 3). Panel B. Full length DOR was fused in frame with the fluorescent protein GFP. To determine whether DOR-GFP retained the capacity to coactivate TR_α1_, experiments were done as in Figure 3A. * significant difference compared to the nuclear hormone receptor group, at P<0.05 (post hoc *t* test). Panel C. HeLa cells were transiently cotransfected with DOR-GFP and TR_α1 _expression vectors. The intranuclear positioning of DOR relative to PML nuclear bodies and TR_α1 _was determined before and after a range of times after T_3_ addition. Antibodies and immunofluorescence legend: Anti-DOR, stained red (column 1); anti-PML, stained green (column 2), anti-TR_α1_ cyan. Merged images: DOR/PML (column 3), DOR/TR_α1_ (column 4).

Current models propose that key components of transcriptional complexes are functionally compartmentalized [35], [36] so that the achievement of a transcriptionally active status implies physical recruitment of chromatin and related proteins. Given that DOR is localized in PML nuclear bodies, and that it functionally activates TR in the presence of T_3_, we aimed to determine whether DOR positioning in PML was affected by the presence of ligands. In cells over-expressing both TR_α1_ and DOR, the addition of T_3 _caused the intranuclear movement of DOR protein from its basal position in PML nuclear bodies (Figure 8A). These effects were not detected in cells that over-expressed only DOR (Figure 8A). To gain further insight into the kinetics of the process, a DOR-GFP construct was generated and transfected in HeLa cells. The chimeric DOR-GFP protein retained the capacity to stimulate the transcriptional activity of TR_ α1_ (Figure 8B) compared to the activity of wild-type DOR. Immunolocalization analysis indicated that DOR-GFP rapidly moved after exposure to T_3_ (already detectable at 5 min) (Figure 8C); the effects were transient and after 60 min of treatment with T_3_, the extent of colocalization of DOR and PML was similar to that detected in basal cells (Figure 8C). Further time-lapse studies indicated that T_3_ caused a rapid change in the localization of DOR-GFP (detectable in less than 1 min) in HeLa cells (data not shown).

Given these findings, we postulate that DOR is localized in PML nuclear bodies mainly as a storage site in which it remains until required. In this regard, TR-mediated responses trigger the mobilization of DOR from the PML bodies. The sensitivity of DOR to T_3_ reinforces the notion that the cellular role of DOR is linked to the regulation of TR function.

## Discussion

Here we have identified a novel protein, named DOR, by means of a substractive hybridization screening aimed to detect genes down-regulated in skeletal muscle from ZDF rats. *DOR* was abundantly expressed in tissues with high metabolic rates such as skeletal muscle and heart. The experimentally induced DOR repression in muscle cells (via siRNA) markedly reduced the action of thyroid hormones and altered muscle differentiation. In this regard, it has been reported that type 2 diabetes is characterized by reduced thyroid function [37]–[39]. In addition, skeletal muscle atrophy is a well-documented complication of diabetes and is characterized by a reduction in the diameter of myofibers and a decreased number of myonuclei [27]–[30]. All these data, together with our observation of a marked reduction of *DOR* expression in skeletal muscle from ZDF rats, lead us to propose that DOR participates in the pathophysiology of type 2 diabetes.

We observed that DOR resides in PML nuclear bodies and shows several properties characteristics of nuclear co-activators. Thus, DOR moderately enhanced the transcriptional activity (2.5- to 5-fold) of TRs in a ligand-dependent manner and acted as an activator when tethered to DNA. In addition, DOR bound to TRs *in vitro* and *in vivo* conditions and to the thyroid hormone responsive *dio1* promoter, as shown by ChIP. The transcriptional activation capacity of DOR occurred through the N-half of the protein, and deletion of its C-terminal half further increased its activity. Whether DOR is a bona fide nuclear co-activator and whether it exerts additional cellular roles remains to be elucidated.

More specifically, we have demonstrated that DOR participates in thyroid hormone action. The supporting evidence is as follows: a) DOR over-expression enhances the transcriptional activity of TR_α1 _4-fold, b) DOR loss-of-function represses the stimulatory effect of thyroid hormones on the expression of genes such as *actin α1*, *caveolin-3*, *creatine kinase*, *IGF-II*, *UCP2* and *myogenin* in muscle cells. c) DOR binds to TR_α1_ *in vitro* and *in vivo* in the context of a T_3_-responsive promoter (human *dio1* promoter), and d) DOR undergoes a rapid and transient intranuclear movement from PML nuclear bodies in response to T_3_. The rapid changes in the nuclear localization of DOR in response to T_3_, may be relevant in the ligand-dependent DOR-mediated potentiation of TR_α1 _activity.

DOR protein contains two functionally distinct regions. The N-terminal half with predicted random structure (GLOBPLOT 2) shows transcriptional activation capacity (mapped between amino acid residues 31 to 111). In this regard, DOR may belong to the group of proteins characterized by sizeable regions that lack a predicted well-structured three-dimensional fold, which show high conservation among species (from mouse to human in the case of DOR) and in which, contrary to the traditional view, the disordered region is functional [40]. The C-terminal region of DOR is predicted to form a positively charged alpha-helix structure and has no transcriptional activation capacity; in contrast, the presence of the C-terminal region reduces the transcriptional activity of the N-terminal half. This observation supports the notion that the transcriptional activity of DOR is subjected to intramolecular control. Such control of transcriptional activity has been reported for other nuclear proteins such as ATF2 or NK-2 [41], [42].

Thyroid hormones stimulate muscle development and differentiation [8] as well as myogenin, and myotube formation in muscle cells [9]–[12], [43]. Moreover, these hormones induce the expression of muscle-specific genes such as *α-actin* and *GLUT4* [5]–[7]. We have demonstrated that in C2C12 muscle cells thyroid hormones also potently stimulate the expression of other genes such as *caveolin-3*, *creatine kinase*, *IGF-II* and *UCP2*. The induction of *IGF-II* may be particularly relevant since it modulates the biology of muscle cells [44]. In addition, and more central to our study, we have found that DOR loss-of-function markedly reduced the myogenic effect of thyroid hormones in muscle cells, as assessed by the expression *myogenin*, *α-actin*, *caveolin-3*, *creatine kinase*, *IGF-II* and *UCP-2*. Thus, our data implicate DOR in the specific stimulatory effects of thyroid hormones on muscle differentiation.

In fact, DOR loss-of-function also affected the capacity of myoblasts to undergo myogenesis. C2C12 muscle KD cells for DOR showed a lower induction of *myogenin* expression, and a reduced expression of *creatine kinase*, *α-actin* and *caveolin-3*. These results indicate that DOR regulates muscle differentiation, at least in part, by controlling *myogenin* expression.

On the basis of our findings, we propose that *DOR* repression participates in a deficient response of muscle to thyroid hormones and in the alterations of muscle biology associated with the diabetic condition.

## Materials and Methods

### Animals

Two month-old male Zucker diabetic fatty rats (ZDF) rats and non-diabetic lean (+/?) controls were purchased from Charles River Laboratories (Wilmington, MA). The animals were housed in animal quarters at 22°C with a 12 h light/12 h dark cycle and fed *ad libitum*. On the experimental day, rats were anesthesized with sodium pentobarbital and gastrocnemius muscles of non-diabetic lean and ZDF rats were collected. All procedures were approved by the Animal Ethics Committee of the University of Barcelona.

### Subtractive hybridization and cDNA cloning

Messenger RNA was extracted from *gastrocnemius* muscle of non-diabetic lean and ZDF rats with oligo(dT)20-cellulose columns, as described [45]. Complementary DNA was prepared from 2 µg of mRNA using Superscript II (Life Technologies). PCR-Select cDNA Subtraction kit (Clontech) was used to select genes that are down-regulated in diabetic muscle [45]. The C42 260 bp fragment obtained from subtractive hybridization was used to screen a human heart λ-ZAP cDNA library (Stratagene). Five clones were isolated, one of which contained the full-length cDNA of human *DOR*. This cDNA clone was subcloned and the sequence of human *DOR* was obtained by sequencing both strands with a two-fold coverage minimum. To determine the murine 5′-cDNA sequence, a cDNA clone (AI95670R) covering 1.8 kb was sequenced. The 3′-cDNA was obtained by RT-PCR amplification. The rat *DOR* cDNA 5′-region was obtained by RT-PCR using heterologous primers from the mouse *DOR* sequence. GenBank accession numbers are AJ297792 Homo sapiens mRNA for DOR protein; AJ297793 Mus musculus mRNA for DOR protein; AJ297794 Rattus norvegicus partial mRNA for DOR protein. Mutated versions of *DOR* were generated by the Quick Change Site Directed Mutagenesis Kit (Stratagene). Full-length DOR cDNA, and cDNA fragments encompassing distinct amino acid fragments were PCR-amplified and cloned in the pGBKT7 vector containing the DNA-binding domain of GAL4 (Clontech) and then cloned in pCDNA3.

### RNA expression studies

Total RNA extraction and treatment with DNase I were performed with Rneasy mini kit (Qiagen). Total RNA from tissue samples or from cells was stored at –80°C until further assay. RNA concentration was determined by spectrophotometry at an absorbance of 260 nm. Northern blot assays on 20 µg of total RNA or with human polyA^+^-RNA obtained from several tissues (Human 12-Lane MTN Blot, Clontech) were performed as described [46] using the ^32^P-labelled C42 cDNA fragment or a 0.5 kb cDNA labelled fragment of human glycerol-3-phosphate dehydrogenase (as a control). The C42 rat cDNA fragment is homologous to the nucleotide sequence 2,912–3,172 of human AJ297792 (GenBank). Real-time PCR was performed from 0.1 µg of total RNA from muscle cells, as described [47]. Cyclophilin or HPRT mRNA were assayed as controls in real-time PCR assays.

### Western blot

A rabbit antibody against the DOR-specific peptide PPPAPSLMDESWFVTPPAC (amino acid residues 63–81) was purchased from Research Genetics. Anti-β-actin antibodies were used as a control of loading. Proteins from total homogenates or fractions enriched in nuclear proteins were resolved in 10% SDS-PAGE and transferred to Immobilon sheets. Incubation with antibodies and ECL detection were performed as described [48].

### Cellular localization studies

The full cDNA sequence of human DOR was amplified by PCR and cloned into the HindIII-BamHI sites of the pCDNA3 vector (Invitrogen). Murine cDNA was amplified by PCR and cloned into the pGEM-T Easy vector (Invitrogen). Recombinant GFP-DOR vectors were generated by cloning a PCR product spanning the murine *DOR*-ORF in-frame into the EcoRI and SalI sites of the pEGFP-C2 vector (Clontech). HeLa cells were transfected with the DOR expression vectors by the calcium phosphate precipitation method. In some studies, 36-h transfected cells were fixed with 3% paraformaldehyde and subjected to immunofluorescence microscopy with a confocal scanning microscope (Leica TCS SP2, Leica Lasertechnik GmbH, Manheim, Germany). No bleed-through was detected between channels. Samples were scanned using a 63x Leitz objective (oil) and a zoom ranging from 2.5 to 4 to analyse intracellular regions. The fluorochromes used (Hoestch, Oregon Green or GFP, Alexa-Fluor 546 and Cyanine 5) were excited with UV, 488, 543 and 633 laser lines, respectively. To avoid bleed-through effects in double or triple staining experiments, each dye was scanned independently.

In some experiments, nuclear extracts from transfected cells were obtained as reported [49] and subjected to Western blot analysis with a specific anti-DOR antibody.

### Cell cultures and transcriptional activation assays

HeLa, L6E9, 1C9, CH310T1/2 or C2C12 cells were maintained in DMEM supplemented with 10% FBS, penicillin (100 U/ml), and streptomycin (100 µg/ml). For transient transfection assays, cells were typically plated onto 24-well plates 24 h prior to transfection by the Lipofectamine 2000 method (Invitrogen) as reported [7]. All transient transfections included 10% of the total DNA of expression vector for GFP (pEGFP, Clontech) to normalize for transfection efficiency. In a typical experiment, 150 ng of reporter plasmid, 75 ng of nuclear receptor expression plasmid and 100 to 300 ng of DOR expression vector was transfected. Ligands were dissolved in absolute ethanol (1 µM dexamethasone) or water (1 µM rosiglitazone or 100 nM T_3_). Sixteen hours after transfection, cells were harvested and cell extracts were analyzed for CAT expression by specific CAT-Elisa® kit (Roche) or luciferase assay system (Promega). Transfection efficiency was analyzed by flow cytometric analysis of GFP expression.

The reporter vector used to assay TR activation was as previously described [7], and consists of a functional TR element from the muscle-specific GLUT4 enhancer, cloned at 5′ of a thymidine kinase basal promoter, controlling the expression of the CAT reporter gene (TKCAT). An expression vector for the rat TR_α1_ was also as previously described [7]. To express murine DOR ectopically in cell lines, a PCR fragment spanning the murine ORF was cloned into the EcoRV and SalI sites of the pcDNA3 (Invitrogen) vector. A mutated version of *DOR* (mutDOR) was generated by the Quick Change Site Directed Mutagenesis Kit (Stratagene). Full-length DOR cDNA, and cDNA fragments encompassing amino acid residues 1–120, 120–220 and 31–111 were PCR-amplified and cloned with NdeI and BamHI in the pGBKT7 vector containing the DNA binding domain of GAL4 (Clontech) and subsequently cloned in pCDNA3. The fragment of DOR cDNA encompassing amino acid residues 1–111 was obtained by mutagenesis from construct 1–120 by generating a stop codon at position G112.

### Protein binding assays

Full-length *DOR* with a histidine-tagged N-terminus (DOR-His) was generated. The DOR-His and TRα1-GST fusion proteins were expressed and purified from *E.coli* on affinity beads. Two µg of extract GST or TRα1-GST and 2 µg of DOR-His were incubated in resuspension buffer (10 mM Tris/HCl, 200 mM NaCl, EDTA 0.2% pH 7.5 containing 10 mM PMSF,10 mM aprotinin, 1 mM pepstatin and 1 mM leupeptin). Proteins were incubated with glutathione-Sepharose beads (Pharmacia) for 1 h at 4°C. The beads were then washed three times in 0.5 ml of resuspension buffer in the presence of 0.1 mM Mg_2_Cl. Proteins were eluted in 200 µl of Laemmli sample buffer and subjected to SDS-PAGE. Proteins were then blotted.

The DOR-His and the TRα1 expression vectors were transiently transfected in HeLa cells. Thirty-six hours after transfection, cells were exposed to T_3_ for 1 h or were left untreated. Cells were then rinsed twice with ice-cold PBS containing 0.5 mM PMSF and cytosolic and nuclear fractions were obtained as described [49]. The nuclear soluble fraction was immunoprecipitated by means of a NI-NTA resin (Qiagen) [50]. The immunocomplexes were resolved by SDS-PAGE and Western blot.

### Chromatin immunoprecipitation (ChIP)

DOR and TRα1 expression vectors were transiently transfected in HeLa cells. Thirty-six hours later, cells were exposed to T_3 _for 1 h or left untreated. They were then treated with the cross-linking agent formaldehyde and lysed. Chromatin was then sheared. Immunoprecipitation was performed with antibodies against TRα1, DOR or SRC-1. After ChIP, DNA was purified by phenol/chloroform extraction. Input (1% of total immunoprecipitated) and immunoprecipitated DNA were subjected to PCR analysis with primers flanking the TRE site on the promoter (*dio 1* promoter) (see primer sequences in supplementary methods) or flanking a region of *GPDH* or *IL-2*. The following primers were used for amplification of promoter regions: -*dio 1* (forward: 5′-GAGGCCAAGGCGCGGGTAGGTCATCT-3′; reverse: 5′-CCGGGTCAGGGGAAGGAGTCAG-3′); -*glycerol-3-phosphate dehydrogenase* (*GPDH*) (forward: 5′-GCTCCAATTCCCCATCTCAG-3′; reverse: 5′-CCAGGCTCAGCCAGTCCCAG-3′); -*interleukin-2* (*IL-2*) (forward: 5′-GTTCAGTGTAGTTTTAGGAC-3′; reverse: 5′-CTCTTCTGATGACTCTTTG-3′).

### Lentiviral infection and siRNA generation

DOR siRNA was obtained from sFold software (http://sfold.wadsworth.org). Scrambled siRNA was obtained by scrambling a functional DOR siRNA sequence. Lentiviruses encoding scrambled or DOR siRNA were used as reported [51]. All HIV-1 derived lentiviral constructs (pLVTHM transfer vector, pCMVΔ8,74 helper packaging construct and pMD2G vector encoding for envelope protein) were kindly provided by Dr. Didier Trono from the *Ecole Polytechnique Federale de Lausanne* (Switzerland) and used as reported [51]. The pLVTHM vector contains a GFP expression cassette and two restriction sites (*ClaI* and *MluI*) after the H1 promoter, thereby allowing direct siRNA cloning. Lentiviruses encoding scrambled and DOR siRNA were produced by triple transient transfection of HEK 293T cells using the calcium-phosphate method. Subconfluent cells were transfected with 10 µg of pLVTHM encoding scrambled or DOR siRNA, 7 µg of pCMVΔ8,74 and 3 µg of pMD2G. Culture medium containing lentiviruses was harvested 48 and 72 h after transfection. Lentiviruses were concentrated by ultracentrifugation (26,000 rpm, 1 h 30 min at 4°C, using a 4 ml sucrose 20% cushion) and resuspended in 100 µl fresh medium. We stored lentiviral aliquots at −80°C. Titration was performed transducing 10^5^ HEK293T cells grown in 12-well plates with 1, 10 or 100 µl of a 1/100 dilution of the concentrated lentiviruses. After 48 h, the percentage of transduced HEK 293T cells (% GFP positive cells) was determined using an EPIC ® S XL flow cytometer (Beckman Coulter ®).

Fifteen million C2C12 myoblasts grown on 12-well plates were transduced at moi 100 and cells were amplified during 5–7 days. Transduced cells (GFP-positive) were then sorted with a MoFlo® flow cytometer (DakoCytomation®, Summit v 3.1 software), obtaining between 93%–99% GFP-positive cells.

### Statistical analysis

Data are presented as means±SD. An unpaired Student's *t* test was used to compare two groups. When experimental series involved more than two groups, statistical analysis was done by one-way analysis of variance or two-way analysis of variance and further post hoc Dunnett's or Tukey's *t* tests. Statistical analyses were performed using the Graph Prism programme (GraphPad Software).
